# *Notes from the Field:* Tobacco Product Use Among Adults — United States, 2017–2023

**DOI:** 10.15585/mmwr.mm7407a3

**Published:** 2025-03-06

**Authors:** René A. Arrazola, Corinne G. Husten, Monica E. Cornelius, Brian S. Armour

**Affiliations:** ^1^Office on Smoking and Health, National Center for Chronic Disease Prevention and Health Promotion, CDC; ^2^Katmai Government Services, Anchorage, Alaska.

**Summary**

**What is already known about this topic?**

Although adult cigarette smoking prevalence remains at its lowest level in 60 years, tobacco use is the leading cause of preventable death in the United States. Change in use of other commercial tobacco products by U.S. adults affects overall tobacco use.

**What is added by this report?**

During 2017–2023, the approximate 6.8 million-person decrease in the number of adults who currently exclusively smoke cigarettes was offset by an approximate 7.2 million-person increase in the number who currently exclusively use e-cigarettes.

**What are the implications for public health practice?**

While current cigarette smoking has decreased to the lowest level in 60 years, current tobacco product use among adults has not changed since 2017. Comprehensive strategies, such as price increases, smoke-free policies, high-impact media campaigns, and cessation support, are critical to preventing and reducing tobacco product use, nicotine addiction, and their associated adverse health outcomes.

Cigarette smoking among U.S. adults has declined from 42.4% in 1965 to 11.6% in 2022 (*1*–*3*); however, tobacco use remains the leading cause of preventable death (*1*,*4*). Current e-cigarette use prevalence among youths increased substantially during 2017–2018 (*4*) but subsequently declined (*5*), and some of these youths are now likely included in surveys of adults. This report describes trends in the use of commercial tobacco products,* including combustible tobacco products, smokeless tobacco products, and e-cigarettes (e-cigarettes meet the federal definition of tobacco products) among adults over a 7-year period and how these trends have affected overall tobacco product use.

## Investigation and Outcomes

CDC analyzed 2017–2023 tobacco product use data among adults aged ≥18 years from the National Health Interview Survey, an annual cross-sectional household survey of the noninstitutionalized U.S. civilian population. Sample sizes ranged from 21,153 (2020) to 31,997 (2019); response rates ranged from 47.0% (2023) to 59.1% (2019).^†^ Current use^§^ of tobacco, overall and by type and exclusivity,^¶^ was assessed overall and by age group (18–24, 25–44, 45–64, and ≥65 years). Weighted prevalences and estimates of the number of persons who use each category of tobacco product (population estimates) and 95% CIs were calculated using SAS-callable SUDAAN software (version 11.0.4; RTI International).** The average annual percent change (AAPC), which measures relative change, in tobacco product use prevalences and population estimates was calculated using the Joinpoint Regression Program (version 5.2.0; National Cancer Institute)^††^ overall and by age group. Linear trends measured by AAPC were considered statistically significant if Benjamini-Hochberg–adjusted p-values were <0.05. This activity was reviewed by CDC, deemed not research, and was conducted consistent with applicable federal law and CDC policy.^§§^

From 2017 to 2023, significant declines in current exclusive cigarette smoking prevalence (10.8% to 7.9% [−5.2 AAPC]) and population estimates (26.6 million to 19.8 million [−5.0 AAPC]) were found, along with increases in exclusive e-cigarette use prevalence (1.2% to 4.1% [20.3 AAPC]) and population estimates (2.9 million to 10.1 million [20.4 AAPC]) (Table). Among adults aged 18–24 years, decreases in prevalences of exclusive cigarette and pipe smoking (6.5% to 1.2% [−21.3 AAPC] and 1.0% to 0.1% [−26.2 AAPC], respectively) and population estimates (1.9 million to 350,000 [−21.5 AAPC] and 290,000 to 40,000 [−25.0 AAPC], respectively) were identified. Within this age group, increases in exclusive e-cigarette use prevalence (2.7% to 10.3% [21.0 AAPC]) and population estimates (800,000 to 3.1 million [21.3 AAPC]) were found. Among adults aged 25–44 years, decreases in prevalence of exclusive cigarette smoking (12.0% to 7.6% [−8.2 AAPC]) and population estimates (10.1 million to 6.5 million [−8.2 AAPC]) were identified, along with increases in exclusive e-cigarette use prevalence (1.5% to 6.1% [24.5 AAPC]) and population estimates (1.3 million to 5.2 million [24.7 AAPC]). Among adults aged 45–64 years, an increase in exclusive e-cigarette use in population estimates (690,000 to 1.6 million [11.5 AAPC]) was identified. Among adults aged ≥65 years, decreases in prevalence of exclusive pipe smoking (0.4% to 0.1% [−17.8 AAPC]) and population estimates (190,000 to 80,000 [−11.4 AAPC]), along with an increase in population estimates of exclusive cigarette smoking (3.6 million to 4.2 million [2.7 AAPC]) were identified.^¶¶^

**TABLE:** Trends in percentage and number of U.S. adults reporting types of current commercial tobacco* product use, by age and product use pattern — National Health Interview Survey, United States, 2017–2023

Age group/Current tobacco or nicotine-containing product use	Prevalence				Estimated no.^†^ of persons who use tobacco products		
	2017	2023	AAPC (95% CI)	2017		2023	AAPC (95% CI)
	% (95% CI)	% (95% CI)		No. (95% CI)		No. (95% CI)	
**All age groups**							
Any tobacco product use^§^	19.3 (18.6 to 20.0)	19.5 (18.9 to 20.2)	−0.3 (–2.3 to 1.9)	474.3 (452.3 to 496.3)		485.9 (465.0 to 506.9)	−0.1 (−2.8 to 2.5)
Exclusive cigarette smoking^¶^	10.8 (10.3 to 11.3)	7.9 (7.5 to 8.4)	–5.2 (–7.2 to –3.2)**	265.9 (251.7 to 280.0)		197.9 (186.4 to 209.5)	–5.0 (−6.9 to −3.1)**
Exclusive cigar smoking^††^	1.9 (1.7 to 2.1)	2.0 (1.8 to 2.2)	0.5 (–2.3 to 3.5)	46.7 (40.9 to 52.5)		49.7 (44.6 to 54.8)	0.6 (−2.7 to 4.1)
Exclusive pipe smoking^§§^	0.4 (0.3 to 0.5)	0.3 (0.2 to 0.3)	–0.8 (–6.0 to 5.2)	9.7 (7.1 to 12.2)		6.8 (5.2 to 8.3)	–2.1 (−11.7 to 9.2)
Exclusive e-cigarette use^¶¶^	1.2 (1.0 to 1.4)	4.1 (3.7 to 4.4)	20.3 (14.0 to 28.9)**	29.1 (24.3 to 34.0)		101.2 (92.5 to 110.0)	20.4 (14.1 to 28.7)**
Exclusive smokeless tobacco use***	1.3 (1.2 to 1.5)	1.4 (1.2 to 1.5)	−1.3 (−5.9 to 3.6)	32.8 (28.5 to 37.0)		33.7 (29.6 to 37.8)	−1.5 (−5.5 to 2.6)
Two or more tobacco product use (cigarette and e-cigarette combinations)^†††^	1.4 (1.2 to 1.6)	1.8 (1.6 to 2.0)	3.7 (−3.4 to 11.9)	34.1 (30.0 to 38.2)		45.1 (39.9 to 50.2)	3.9 (−3.7 to 12.7)
Two or more tobacco product use (other product combinations)^§§§^	2.3 (2.0 to 2.5)	2.1 (1.9 to 2.3)	−1.8 (−4.4 to 1.0)	55.8 (49.4 to 62.2)		51.3 (45.8 to 56.7)	−1.8 (−5.8 to 2.4)
**18–24 yrs**							
Any tobacco product use^§^	18.3 (16.3 to 20.4)	16.8 (14.9 to 18.9)	−0.2 (−3.7 to 3.6)	53.6 (46.7 to 60.5)		50.1 (43.5 to 56.8)	−0.2 (−3.5 to 3.2)
Exclusive cigarette smoking^¶^	6.5 (5.3 to 7.9)	1.2 (0.7 to 1.9)	–21.3 (−26.2 to −16.1)**	19.0 (15.1 to 22.9)		3.5 (1.9 to 5.1)	−21.5 (−25.9 to −16.8)**
Exclusive cigar smoking^††^	1.4 (1.0 to 2.1)	1.0 (0.6 to 1.7)	−6.5 (−15.8 to 3.0)	4.2 (2.5 to 5.9)		2.9 (1.3 to 4.6)	−6.9 (−13.0 to −0.9)
Exclusive pipe smoking^§§^	1.0 (0.6 to 1.6)	0.1 (0.0 to 0.4)	−26.2 (−41.5 to −14.9)**	2.9 (1.5 to 4.3)		0.4 (0.0 to 0.9)	−25.0 (−39.9 to −12.5)**
Exclusive e-cigarette use^¶¶^	2.7 (1.9 to 4.0)	10.3 (8.7 to 12.0)	21.0 (8.1 to 42.1)**	8.0 (4.9 to 11.1)		30.6 (25.4 to 35.8)	21.3 (9.7 to 39.2)**
Exclusive smokeless tobacco use***	1.4 (0.9 to 2.2)	0.3 (0.1 to 0.8)	−18.7 (−37.2 to −2.3)	4.2 (2.3 to 6.0)		0.9 (0.0 to 1.8)	−19.8 (−39.3 to −1.9)
Two or more tobacco product use (cigarette and e-cigarette combinations)^†††^	1.7 (1.2 to 2.3)	1.8 (1.3 to 2.5)	2.0 (−5.6 to 10.6)	4.9 (3.2 to 6.5)		5.3 (3.4 to 7.1)	2.2 (−5.2 to 10.6)
Two or more tobacco product use (other product combinations)^§§§^	3.5 (2.7 to 4.5)	2.1 (1.5 to 3.0)	−5.8 (−13.2 to 1.6)	10.3 (7.6 to 12.9)		6.3 (4.0 to 8.5)	−5.9 (−13.3 to 1.6)
**25–44 yrs**							
Any tobacco product use^§^	22.5 (21.4 to 23.7)	24.4 (23.2 to 25.6)	0.3 (−2.3 to 3.0)	189.1 (177.2 to 177.2)		206.4 (194.6 to 218.2)	0.4 (−2.2 to 3.1)
Exclusive cigarette smoking^¶^	12.0 (11.2 to 12.9)	7.6 (7.0 to 8.3)	−8.2 (−11.5 to −4.7)**	101.0 (93.1 to 108.9)		64.5 (58.4 to 70.7)	−8.2 (−11.4 to −4.9)**
Exclusive cigar smoking^††^	2.4 (2.1 to 2.9)	2.6 (2.2 to 3.0)	1.4 (−2.2 to 5.1)	20.5 (16.8 to 24.2)		21.6 (18.0 to 25.1)	0.8 (−2.6 to 4.3)
Exclusive pipe smoking^§§^	0.4 (0.2 to 0.6)	0.6 (0.4 to 0.7)	8.8 (−1.8 to 20.4)	3.1 (1.6 to 4.7)		4.6 (3.3 to 5.9)	8.1 (−2.5 to 19.8)
Exclusive e-cigarette use^¶¶^	1.5 (1.2 to 1.9)	6.1 (5.5 to 6.8)	24.5 (19.5 to 31.2)**	12.5 (9.7 to 15.2)		51.6 (46.1 to 57.2)	24.7 (19.4 to 32.2)**
Exclusive smokeless tobacco use***	1.4 (1.2 to 1.7)	1.5 (1.3 to 1.8)	−1.5 (−9.6 to 7.9)	11.9 (9.6 to 14.2)		12.8 (10.5 to 15.1)	−1.5 (−8.1 to 5.8)
Two or more tobacco product use (cigarette and e-cigarette combinations)^†††^	1.9 (1.5 to 2.2)	3.0 (2.6 to 3.6)	6.4 (−2.4 to 16.3)	15.5 (12.7 to 18.3)		25.7 (21.5 to 29.9)	7.0 (−2.6 to 18.0)
Two or more tobacco product use (other product combinations)^§§§^	2.9 (2.4 to 3.4)	3.0 (2.6 to 3.5)	−0.9 (−8.7 to 8.3)	24.3 (20.0 to 28.6)		25.2 (21.5 to 29.0)	−0.9 (−9.0 to 8.5)
**45–64 yrs**							
Any tobacco product use^§^	21.3 (20.1 to 22.5)	21.0 (19.9 to 22.1)	−0.6 (−2.1 to 1.0)	177.3 (166.0 to 188.6)		164.0 (153.8 to 174.1)	−1.6 (–3.2 to 0.0)
Exclusive cigarette smoking^¶^	13.2 (12.3 to 14.2)	11.2 (10.4 to 12.1)	−2.6 (−4.8 to −0.4)	110.0 (101.6 to 118.4)		87.7 (80.5 to 95.0)	−3.5 (−7.2 to 0.5)
Exclusive cigar smoking^††^	2.0 (1.7 to 2.4)	2.1 (1.8 to 2.5)	0.6 (−3.7 to 5.4)	16.6 (13.3 to 19.8)		16.5 (13.7 to 19.2)	−0.1 (−4.3 to 4.5)
Exclusive pipe smoking^§§^	0.2 (0.1 to 0.3)	0.1 (0.1 to 0.2)	2.6 (−17.6 to 29.1)	1.6 (0.7 to 2.5)		0.9 (0.3 to 1.4)	−1.4 (−16.3 to 16.9)
Exclusive e-cigarette use^¶¶^	0.8 (0.6 to 1.1)	2.0 (1.7 to 2.4)	12.4 (2.8 to 23.0)	6.9 (5.1 to 8.6)		15.7 (13.0 to 18.4)	11.5 (4.3 to 20.8)**
Exclusive smokeless tobacco use***	1.5 (1.3 to 1.9)	2.0 (1.7 to 2.4)	3.5 (−2.7 to 10.1)	12.7 (10.2 to 15.2)		15.5 (12.7 to 18.3)	2.0 (−3.3 to 7.5)
Two or more tobacco product use (cigarette and e-cigarette combinations)^†††^	1.5 (1.2 to 1.8)	1.6 (1.3 to 1.9)	0.8 (−9.3 to 12.1)	12.1 (9.5 to 14.7)		12.4 (10.1 to 14.6)	−0.1 (−10.1 to 11.0)
Two or more tobacco product use (other product combinations)^§§§^	2.1 (1.7 to 2.4)	1.9 (1.6 to 2.3)	−2.3 (−7.4 to 3.0)	17.1 (14.3 to 20.0)		15.1 (12.2 to 17.9)	−2.6 (−8.3 to 3.5)
**≥65 yrs**							
Any tobacco product use^§^	11.0 (10.1 to 11.9)	11.6 (10.9 to 12.4)	0.3 (−1.7 to 2.6)	54.1 (49.5 to 58.8)		65.3 (60.8 to 69.8)	2.4 (−0.1 to 5.3)
Exclusive cigarette smoking^¶^	7.2 (6.5 to 8.1)	7.5 (6.9 to 8.1)	0.7 (−0.7 to 2.3)	35.7 (31.8 to 39.7)		42.0 (38.3 to 45.6)	2.7 (0.9 to 4.8)**
Exclusive cigar smoking^††^	1.1 (0.8 to 1.4)	1.5 (1.3 to 1.8)	3.2 (−3.2 to 10.0)	5.3 (3.9 to 6.7)		8.6 (7.0 to 10.2)	6.0 (−0.1 to 12.6)
Exclusive pipe smoking^§§^	0.4 (0.2 to 0.6)	0.1 (0.1 to 0.3)	−17.8 (−28.2 to −8.4)**	1.9 (1.0 to 2.7)		0.8 (0.3 to 1.2)	−11.4 (−19.1 to −3.3)**
Exclusive e-cigarette use^¶¶^	0.3 (0.2 to 0.5)	0.6 (0.4 to 0.8)	8.0 (−1.4 to 20.2)	1.7 (1.0 to 2.4)		3.1 (2.2 to 4.1)	8.8 (0.4 to 19.1)
Exclusive smokeless tobacco use***	0.8 (0.6 to 1.1)	0.8 (0.6 to 1.0)	−4.0 (−9.1 to 1.7)	3.8 (2.5 to 5.1)		4.4 (3.2 to 5.6)	−2.0 (−8.8 to 6.4)
Two or more tobacco product use (cigarette and e-cigarette combinations)^†††^	0.3 (0.2 to 0.5)	0.3 (0.2 to 0.4)	2.5 (−14.8 to 25.4)	1.5 (0.8 to 2.1)		1.6 (0.9 to 2.3)	2.4 (−10.0 to 16.7)
Two or more tobacco product use (other product combinations)^§§§^	0.8 (0.6 to 1.1)	0.8 (0.6 to 1.1)	−2.9 (−8.2 to 3.0)	4.0 (2.7 to 5.2)		4.5 (3.4 to 5.7)	–0.1 (−4.5 to 4.7)

**Abbreviation: **AAPC = average annual percent change.

* Commercial tobacco refers to tobacco products that are made and sold by companies. This definition does not include traditional tobacco used by some Indigenous groups for religious or ceremonial purposes. In this report, tobacco refers to the following commercial tobacco products: cigarettes, cigars, e-cigarettes, pipes, and smokeless tobacco.

^†^ Multiplied by 100,000 and rounded down to the nearest 10,000.

^§^ Current use of cigarettes, cigars, pipes, e-cigarettes, or smokeless tobacco alone or in any combination.

^¶^ Ever smoked 100 or more cigarettes during one’s lifetime and smoking every day or some days at the time of the survey without use of any other tobacco product.

** Significant Benjamini-Hochberg adjusted p-values with a false discovery rate set at 0.05 for average AAPC different from zero from Joinpoint regression for linear trend.

^††^ Ever smoked a regular cigar, cigarillo, or little filtered cigar during one’s lifetime and reported smoking every day or some days at the time of the survey without use of any other tobacco product.

^§§^ Ever smoked a pipe filled with tobacco (either a regular pipe, water pipe, or hookah) during one’s lifetime and reported smoking every day or some days at the time of survey without use of any other tobacco product.

^¶¶^ Ever use of an e-cigarette or other electronic vaping product during one’s lifetime and reported using such a product every day or some days at the time of the survey without use of any other tobacco product.

*** Ever use of a smokeless tobacco product (chewing tobacco, snuff, dip, snus, or dissolvable tobacco) during one’s lifetime and reported using every day or some days at the time of the survey without use of any other tobacco product.

^†††^ Current use of e-cigarettes and current smoking of cigarettes with or without use of any other tobacco product.

^§§§^ Current use of any other combination of tobacco products without concurrent use of cigarettes and e-cigarettes.

## Preliminary Conclusions and Actions

The decrease in number of adults who currently exclusively smoke cigarettes by approximately 6.8 million persons was offset by the increase in the number who currently use e-cigarettes exclusively (approximately 7.2 million). This increase was primarily driven by increases among adults aged 18–24 and 25–44 years (approximately 2.3 million and 3.9 million, respectively), leading to no net change in overall current adult tobacco product use.

Continued surveillance and use of comprehensive tobacco control strategies, such as price increases, smokefree policies, high-impact media campaigns, and cessation support, are important for preventing and reducing tobacco product use, nicotine addiction, and their associated adverse health outcomes (*1*,*4*).

## Footnotes

* Commercial tobacco refers to tobacco products that are made and sold by companies. This definition does not include traditional tobacco used by some Indigenous groups for religious or ceremonial purposes. In this report, tobacco refers to the following commercial tobacco products: cigarettes, cigars, e-cigarettes, pipes, and smokeless tobacco. E-cigarettes meet the federal definition of tobacco products. https://www.fda.gov/tobacco-products/products-ingredients-components/e-cigarettes-vapes-and-other-electronic-nicotine-delivery-systems-ends

†
https://www.cdc.gov/nchs/nhis/documentation/?CDC_AAref_Val=https://www.cdc.gov/nchs/nhis/data-questionnaires-documentation.htm

§ Current use is defined as reported use of cigarettes (ever smoked 100 or more cigarettes during one’s lifetime); cigar (ever smoked a regular cigar, cigarillo, or little filtered cigar); pipe (regular pipe, water pipe, or hookah filled with tobacco); e-cigarette or other electronic vaping product; smokeless tobacco (chewing tobacco, snuff, dip, snus, or dissolvable tobacco), and using a tobacco product (cigarettes, cigars, pipes, e-cigarettes, or smokeless tobacco, alone or in any combination) “every day” or “some days” at the time of the survey.

¶ Exclusive use is defined as current use of any listed tobacco product alone (without using any other tobacco product); combination use is defined as 1) current use of two or more tobacco products that includes both cigarettes and e-cigarettes, with or without use of any other tobacco product or 2) current use of two or more tobacco products that does not include a combination of cigarettes and e-cigarettes.

** Data were weighted to account for complex survey design and to adjust for nonresponse. https://support.sas.com/resources/papers/proceedings19/3659-2019.pdf

††
https://surveillance.cancer.gov/joinpoint/

§§ 45 C.F.R. part 46.102(l)(2), 21 C.F.R. part 56; 42 U.S.C. Sect. 241(d); 5 U.S.C. Sect. 552a; 44 U.S.C. Sect. 3501 et seq.

¶¶ Caution is warranted when comparing changes during 2019–2023 with earlier years of National Health Interview Survey (NHIS) data because of changes in weighting and design methodology (https://www.cdc.gov/nchs/data/nhis/earlyrelease/EReval202009-508.pdf). NHIS quarterly estimates during 2019–2023 indicate decreases in current cigarette smoking and increases in current e-cigarette use, which is different from what the forecasting models predicted. https://wwwn.cdc.gov/NHISDataQueryTool/ER_Quarterly/index_quarterly.html
